# Effects of blood pressure and tranexamic acid in spontaneous intracerebral haemorrhage: a secondary analysis of a large randomised controlled trial

**DOI:** 10.1136/bmjno-2023-000423

**Published:** 2023-06-12

**Authors:** Jason Philip Appleton, Zhe Kang Law, Lisa Jane Woodhouse, Rustam Al-Shahi Salman, Maia Beridze, Hanne Christensen, Robert A Dineen, Juan José Egea Guerrero, Timothy J England, Michal Karlinski, Kailash Krishnan, Ann Charlotte Laska, Philippe Lyrer, Serefnur Ozturk, Christine Roffe, Ian Roberts, Thompson G Robinson, Polly Scutt, David J Werring, Philip M Bath, Nikola Sprigg

**Affiliations:** 1 Stroke, Nottingham University Hospitals NHS Trust, Nottingham, UK; 2 Stroke Trials Unit, Mental Health and Clinical Neurosciences, University of Nottingham, Nottingham, UK; 3 Neurology Unit, Department of Medicine, National University of Malaysia Faculty of Medicine, Kuala Lumpur, Malaysia; 4 Centre for Clinical Brain Sciences, University of Edinburgh, Edinburgh, UK; 5 The First University Clinic, Tbilisi State Medical University, Tbilisi, Georgia; 6 Department of Neurology, Copenhagen University Hospital, Bispebjerg, Denmark; 7 Radiological Sciences, Mental Health and Clinical Neurosciences, University of Nottingham, Nottingham, UK; 8 NIHR Nottingham Biomedical Research Centre, Nottingham, UK; 9 Neurocritical Care Unit, Virgen del Rocio University Hospital, Sevilla, Spain; 10 IbiS, CSIC, University of Seville, Sevilla, Spain; 11 2nd Department of Neurology, Institute of Psychiatry and Neurology, Warsaw, Poland; 12 Department of Clinical Sciences, Danderyd Hospital, Karolinska Institute, Stockholm, Sweden; 13 Neurology and Stroke Center, University Hospital Basel, Basel, Switzerland; 14 Neurology, Faculty of Medicine, Selcuk Universitesi, Konya, Turkey; 15 Stroke Research, School of Medicine, University of Keele, Stoke-on-Trent, UK; 16 Clinical Trials Unit, London School of Hygiene and Tropical Medicine, London, UK; 17 College of Life Sciences, University of Leicester, Leicester, UK; 18 Stroke Research Centre, Department of Brain Repair and Rehabilitation, UCL Queen Square Institute of Neurology, London, UK

**Keywords:** STROKE

## Abstract

**Background:**

Tranexamic acid reduced haematoma expansion and early death, but did not improve functional outcome in the tranexamic acid for hyperacute spontaneous intracerebral haemorrhage-2 (TICH-2) trial. In a predefined subgroup, there was a statistically significant interaction between prerandomisation baseline systolic blood pressure (SBP) and the effect of tranexamic acid on functional outcome (p=0.019).

**Methods:**

TICH-2 was an international prospective double-blind placebo-controlled randomised trial evaluating intravenous tranexamic acid in patients with acute spontaneous intracerebral haemorrhage (ICH). Prerandomisation baseline SBP was split into predefined ≤170 and >170 mm Hg groups. The primary outcome at day 90 was the modified Rankin Scale (mRS), a measure of dependency, analysed using ordinal logistic regression. Haematoma expansion was defined as an increase in haematoma volume of >33% or >6 mL from baseline to 24 hours. Data are OR or common OR (cOR) with 95% CIs, with significance at p<0.05.

**Results:**

Of 2325 participants in TICH-2, 1152 had baseline SBP≤170 mm Hg and were older, had larger lobar haematomas and were randomised later than 1173 with baseline SBP>170 mm Hg. Tranexamic acid was associated with a favourable shift in mRS at day 90 in those with baseline SBP≤170 mm Hg (cOR 0.73, 95% CI 0.59 to 0.91, p=0.005), but not in those with baseline SBP>170 mm Hg (cOR 1.05, 95% CI 0.85 to 1.30, p=0.63). In those with baseline SBP≤170 mm Hg, tranexamic acid reduced haematoma expansion (OR 0.62, 95% CI 0.47 to 0.82, p=0.001), but not in those with baseline SBP>170 mm Hg (OR 1.02, 95% CI 0.77 to 1.35, p=0.90).

**Conclusions:**

Tranexamic acid was associated with improved clinical and radiological outcomes in ICH patients with baseline SBP≤170 mm Hg. Further research is needed to establish whether certain subgroups may benefit from tranexamic acid in acute ICH.

**Trial registration number:**

ISRCTN93732214.

**WHAT IS ALREADY KNOWN ON THIS TOPIC**

- Tranexamic acid did not improve functional outcome despite reducing haematoma expansion and early death in patients with acute spontaneous intracerebral haemorrhage in the tranexamic acid for hyperacute spontaneous intracerebral haemorrhage-2 trial, but there was a statistically significant interaction between baseline systolic blood pressure (SBP) and treatment on functional outcome, which we sought to explore further.

**WHAT THIS STUDY ADDS**

- In this prespecified secondary analysis, randomisation to tranexamic acid in the presence of baseline SBP ≤170 mm Hg was associated with less haematoma expansion and improved clinical outcomes with fewer deaths and serious adverse events (SAEs), less death and dependency, and improved quality of life scores compared with placebo. A >15% reduction in SBP from baseline to day 2 was associated with fewer deaths by day 7 and 90 in those randomised to tranexamic acid but not placebo, whilst a >5% increase in SBP was associated with increased death and SAEs by day 7 overall, and increased SAEs in those randomised to placebo by days 7 and 90.

**HOW THIS STUDY MIGHT AFFECT RESEARCH, PRACTICE OR POLICY**

- Tranexamic acid may improve clinical and radiological outcomes in participants with baseline SBP≤170 mm Hg. Future research should aim to establish which subgroups of patients may benefit from tranexamic acid and whether BP lowering is additive or synergistic in the presence of tranexamic acid in acute intracerebral haemorrhage.

## Introduction

Elevated blood pressure (BP) in acute intracerebral haemorrhage (ICH) is associated with haematoma expansion and increased death and dependency.^1–3^ Large trials of intensive BP lowering in ICH patients with elevated BP at presentation have produced mixed results: in INTERACT-2 intensive BP lowering was associated with less death and dependency in a shift analysis of the modified Rankin Scale (mRS)^4^; ATACH-2, which assessed a more aggressive intensive BP lowering strategy, did not influence mRS at day 90 but did lead to more renal adverse events.^5^ Current guidelines recommend to consider reduction of elevated BP in acute ICH in line with the INTERACT-2 protocol.^6–8^

In the tranexamic acid for hyperacute spontaneous intracerebral haemorrhage-2 (TICH-2) trial,^9^ tranexamic acid reduced haematoma expansion and early death but did not influence the mRS at day 90. In predefined subgroups, there was a statistically significant interaction between baseline systolic BP (SBP) and tranexamic acid on the primary outcome of mRS at day 90. Those with baseline SBP ≤170 mm Hg randomised to tranexamic acid had a favourable shift in the mRS to less death and dependency compared with placebo, while those with baseline SBP >170 mm Hg randomised to tranexamic acid had no change in the mRS compared with those randomised to placebo.^9^

We sought to investigate this interaction in this predefined subgroup in more detail, and in particular to assess the association of baseline SBP on the potential treatment effect of tranexamic acid. We hypothesised that patients with lower baseline SBP were more likely to have non-hypertension-related ICH aetiologies with more lobar ICH, present later, have milder clinical phenotypes, may not have undergone haematoma expansion, and therefore, might benefit from tranexamic acid.

## Methods

TICH-2 was an international prospective double-blind randomised placebo-controlled clinical trial that tested the safety and efficacy of intravenous tranexamic acid in people with acute spontaneous ICH within 8 hours of symptom onset. Details pertaining to the trial protocol and main results are published.^9 10^ Written consent was obtained from patients or their representatives before starting trial procedures.

### Blood pressure

Baseline BP was measured immediately prior to randomisation and recorded on the randomisation form, a further two BP measurements were taken and recorded on day 2. We used the predefined baseline SBP cut-point used in the statistical analysis plan and main results paper of TICH-2: ≤170 mm Hg and >170 mm Hg.^9 11^ This cut-point was the median baseline SBP found in acute ICH patients previously.^12 13^ We also assessed whether change in SBP from baseline to day 2, independent of baseline SBP, was associated with clinical outcome using categories applied in a previously published secondary analysis of a large acute stroke trial as follows: large decrease (>15% decrease), moderate decrease (5%–15% decrease), no change (5% decrease to 5% increase, reference group), increase (>5% increase).^14^ The association between SBP on day 2 and clinical outcome by treatment group was assessed in those with SBP≤140 mm Hg and >140 mm Hg and in 20 mm Hg increments across the range of day 2 SBP. Data on the number, route and class of antihypertensive medications used between randomisation and day 2 were collected.

### Clinical outcomes

The primary outcome in TICH-2 was functional outcome measured using the mRS by trained assessors over telephone at day 90. Quality of life was recorded at day 90 using European Quality of Life 5-dimensions derived health utility status and European Quality of Life visual analogue scale. Safety outcomes included death and serious adverse events (SAEs) at day 2, day 7, discharge and day 90, and neurological status at day 7 using the National Institute of Health Stroke Scale (NIHSS). In addition, length of hospital stay was recorded.

### Imaging outcomes

A baseline CT brain scan was performed prior to randomisation and a repeat CT scan at 24±12 hours. Haematoma volumes were assessed by three independent raters blinded to clinical data using semiautomated segmentation tools of ITK-SNAP software V.3.6.0.^15^ Haematoma expansion was defined as an increase in haematoma volume on follow-up scan of >33% or >6 mL compared with the baseline scan.^16^

### Statistics

Analyses followed the statistical analysis plan for the overall TICH-2 trial.^11^ Data are number (%), mean (SD), median [IQR]. Baseline characteristics of participants by baseline SBP were compared using X^2^ test, one-way analysis of variance or Kruskal-Wallis test as appropriate. Analyses between treatment groups were assessed by intention to treat. The primary outcome was assessed across all seven levels of the mRS using ordinal logistic regression with adjustment for baseline prognostic variables as in the main TICH-2 trial: age, sex, time since onset to randomisation, baseline SBP, baseline NIHSS, presence of intraventricular haemorrhage and antiplatelet therapy before stroke onset. Sensitivity analyses of the mRS were performed including unadjusted, mRS>3 and with additional adjustment for baseline haematoma volume and location. Other outcomes were analysed using binary logistic, multiple linear or Cox regression models as appropriate with adjustment as outlined above. A sensitivity analysis using >33% increase in haematoma volume to define haematoma expansion was performed, given that absolute volume increase may have a differential effect depending on haematoma location. To assess whether haematoma location influenced the effect of tranexamic acid on the primary outcome, an interaction term was added to an adjusted ordinal logistic regression model. Resultant common OR (cOR), OR, HR or mean difference with corresponding 95% CIs are given, with significance set at p<0.05. Results were not adjusted for multiple testing. Statistical analyses were performed using the SPSS V.23.

## Results

Of the 2325 participants in TICH-2, 1152 had baseline SBP≤170 mm Hg and 1173 baseline SBP>170 mm Hg. Baseline characteristics are depicted in table 1. Those with a baseline SBP≤170 mm Hg were more likely to be older, male, have a previous stroke or TIA, ICH, taking prior antiplatelet and statin therapies, and have less premorbid hypertension. As expected, those with baseline SBP≤170 mm Hg also had lower diastolic BP at baseline than those with baseline SBP>170 mm Hg. Participants with baseline SBP≤170 mm Hg had a longer time from onset to randomisation, more supratentorial lobar haemorrhages than deep haemorrhages, larger baseline haematoma volumes and were less likely to have intraventricular haemorrhage compared with those with baseline SBP>170 mm Hg. CT angiography was performed more frequently in those with baseline SBP≤170 mm Hg, but a positive spot sign—although uncommon—was seen more often in those with baseline SBP>170 mm Hg.

**Table 1:** Baseline characteristics of TICH-2 participants by prerandomisation baseline SBP

	All	SBP≤170 mm Hg	SBP>170 mm Hg	P value
No of patients	2325	1152	1173	
Age (years)	68.9 (13.8)	70 (13.2)	67.8 (14.3)	<0.001
>70	1164 (50.1)	620 (53.8)	544 (46.4)	<0.001
Sex, male (%)	1301 (56)	670 (58.2)	631 (53.8)	0.034
Baseline mRS [/6]	0 [0, 1]	0 [0, 1]	0 [0, 1]	0.057
Medical history (%)				
Hypertension	1421 (61.1)	652 (56.6)	769 (65.6)	<0.001
Diabetes mellitus	312 (13.4)	156 (13.5)	156 (13.3)	0.72
Atrial fibrillation	71 (3.1)	41 (3.6)	30 (2.6)	0.37
Previous stroke/TIA	329 (14.2)	182 (15.8)	147 (12.5)	0.049
Previous ICH	126 (5.4)	84 (7.3)	42 (3.6)	<0.001
Ischaemic heart disease	202 (8.7)	102 (8.9)	100 (8.5)	0.78
Antiplatelet therapy	611 (26.3)	336 (29.2)	275 (23.4)	0.005
Statin therapy	622 (26.8)	348 (30.2)	274 (23.4)	0.001
Qualifying event				
NIHSS [/42]	12 [7,19]	12 [6,19]	13 [7,18]	0.27
GCS [/15]	15 [12,15]	15 [12,15]	14 [12,15]	0.65
Haemodynamics				
BP, systolic (mm Hg) [range]	172.6 (27.1) [98–265]	150.8 (14.3) [98–170]	194 (18.3) [171–265]	<0.001
BP, diastolic (mm Hg) [range]	93.2 (18.1) [36–179]	84.1 (13.6) [36–152]	102.1 (17.5) [55–179]	<0.001
Randomisation to tranexamic acid (%)	1161 (49.9)	590 (51.2)	571 (48.7)	0.22
Time to randomisation [hours]	3.6 [2.6,5]	3.9 [2.8,5.4]	3.4 [2.5,4.7]	<0.001
<3 (%)	833 (35.8)	352 (30.6)	481 (41)	<0.001
<4.5 (%)	1575 (67.7)	726 (63)	849 (72.4)	<0.001
Haematoma location (%)				<0.001
Supratentorial lobar	738 (31.7)	447 (38.8)	291 (24.8)	
Supratentorial deep	1371 (59)	607 (52.7)	764 (65.1)	
Infratentorial	149 (6.4)	65 (5.6)	84 (7.2)	
Combination	67 (2.9)	33 (2.9)	34 (2.9)	
Haematoma volume (mL) [IQR]	13.3 [5.5–32.3]	14.1 [5.7–36.3]	12.5 [5.3–29.5]	0.032
Intraventricular haemorrhage (%)	745 (32)	341 (29.6)	404 (34.4)	0.012
CT angiography done (%)	249 (10.7)	129 (11.2)	120 (10.2)	0.031
Spot sign positive	56 (2.4)	21 (1.8)	35 (3)	0.039

Data are number (%), mean (SD), median [IQR], [range]; comparison across BP by χ^2^ test, Kruskal-Wallis test or one-way ANOVA.

ANOVA, analysis of variance; BP, blood pressure; GCS, Glasgow Coma Score; ICH, intracerebral haemorrhage; mRS, modified Rankin Scale; NIHSS, National Institutes for Health Stroke Scale; SBP, systolic BP; TIA, transient ischaemic attack; TICH-2, tranexamic acid for hyperacute spontaneous intracerebral haemorrhage-2.

Investigator-reported final diagnosis differed by baseline SBP (online supplemental table 1). More participants with ICH secondary to cerebral amyloid angiopathy (CAA) had a baseline SBP≤170 mm Hg compared with SBP>170 mm Hg: 58 (5%) vs 22 (1.9%), p<0.001. Hypertensive arteriopathy was the most common cause of ICH in both baseline groups and more common in those with baseline SBP>170 mm Hg than SBP≤170 mm Hg: 672 (57.3%) vs 449 (39%), p<0.001 (online supplemental table 1).

10.1136/bmjno-2023-000423.supp1 Supplementary data

### Outcomes by baseline SBP

The interaction between baseline SBP and treatment with tranexamic acid on mRS at day 90 was statistically significant, p=0.019. In participants with baseline SBP≤170 mm Hg, randomisation to tranexamic acid was associated with a smaller increase in haematoma volume from baseline to 24 hours and less haematoma expansion than those randomised to placebo (table 2). At day 7, NIHSS scores were lower in those randomised to tranexamic acid compared with those allocated to placebo. There were fewer deaths by days 2, 7, 90 and discharge from hospital in the tranexamic acid group. Further, there were fewer SAEs throughout the trial in those randomised to tranexamic acid; a finding driven by a reduction in nervous system-related SAEs (online supplemental table 2).

**Table 2:** Outcomes by randomised treatment group (tranexamic acid vs placebo) split by prerandomisation baseline SBP

SBP≤170 mm Hg	Tranexamic acid	Placebo	cOR/OR/HR/MD (95% CI)	P value
N (%)	590	562		
Haematoma				
Change in volume from baseline to 24 hours,* mL	3.3 (15.9)	5.1 (15.8)	−1.99 (−3.90, to 0.08)	**0.041**
Haematoma expansion (%)	132 (24.3)	161 (32.5)	0.62 (0.47, 0.82)	**0.001**
Haematoma expansion >33% (%)	101 (18.6)	119 (24)	0.69 (0.51, 0.94)	**0.019**
Day 2				
Death (%)	15 (2.5)	33 (5.9)	0.35 (0.18, 0.68)	**0.002**
SAEs (%)	175 (29.7)	215 (38.3)	0.62 (0.48, 0.81)	**<0.001**
Day 7				
NIHSS	9.8 (7.9)	10.6 (8.9)	−1.21 (−1.92, to 0.51)	**0.001**
Death (%)	43 (7.3)	64 (11.4)	0.53 (0.34, 0.83)	**0.006**
SAEs (%)	217 (36.8)	247 (44)	0.67 (0.52, 0.87)	**0.003**
Day 90				
Primary outcome				
mRS [/6]	4 [2,5]	4 [2,5]	0.73 (0.59, 0.91)	**0.005**
Sensitivity analysis				
mRS, unadjusted	4 [2,5]	4 [2,5]	0.85 (0.69, 1.04)	**0.13**
mRS, adjusted for HV	4 [2,5]	4 [2,5]	0.78 (0.63, 0.97)	**0.025**
mRS, adjusted for HV and location^~^	4 [2,5]	4 [2,5]	0.76 (0.61, 0.94)	**0.012**
mRS>3 (%)	414 (70.2)	418 (74.4)	0.66 (0.48, 0.90)	**0.010**
Death (%)	116 (19.7)	130 (23.1)	0.74 (0.58, 0.95)	**0.020**
SAEs (%)	258 (43.7)	279 (49.6)	0.72 (0.55, 0.93)	**0.012**
EQ-5D HUS (/1)	0.35 (0.4)	0.32 (0.4)	0.04 (0.0, 0.08)	**0.046**
EQ-VAS (/100)	49.5 (33.2)	46.7 (33.1)	3.53 (0.20, 6.86)	**0.038**
Discharge				
Length of hospital stay, days	63.7 (46.7)	63.8 (47.7)	2.08 (−3.30, 7.46)	0.45
Death by discharge (%)	87 (14.7)	107 (19)	0.67 (0.47, 0.96)	**0.030**
SBP>170 mm Hg				
N (%)	571	602		
Haematoma				
Change in volume from baseline to 24 hours,* mL	4.2 (15.8)	4.7 (16.1)	−0.89 (−2.78, 1.00)	0.36
Haematoma expansion (%)	133 (26.1)	143 (25.4)	1.02 (0.77, 1.35)	0.90
Haematoma expansion >33% (%)	101 (19.8)	125 (22.2)	0.84 (0.63, 1.14)	0.27
Day 2				
Death (%)	25 (4.4)	24 (4)	1.01 (0.55, 1.84)	0.99
SAEs (%)	204 (35.7)	202 (33.6)	1.05 (0.81, 1.36)	0.71
Day 7				
NIHSS	10.5 (8.7)	10 (8.4)	0.32 (−0.43, 1.08)	0.40
Death (%)	58 (10.2)	59 (9.8)	0.94 (0.62, 1.42)	0.76
SAEs (%)	239 (41.9)	250 (41.5)	0.95 (0.73, 1.22)	0.67
Day 90				
Primary outcome				
mRS at day 90 [/6]	4 [2,5]	4 [2,5]	1.05 (0.85, 1.30)	0.63
Sensitivity analysis				
mRS at day 90, unadjusted	4 [2,5]	4 [2,5]	1.15 (0.94, 1.41)	0.16
mRS, adjusted for baseline HV	4 [2,5]	4 [2,5]	0.99 (0.79, 1.22)	0.90
mRS, adjusted for baseline HV and location	4 [2,5]	4 [2,5]	1.00 (0.80, 1.24)	0.99
mRS>3 (%)	409 (71.6)	414 (68.8)	1.09 (0.79, 1.51)	0.59
Death (%)	134 (23.5)	119 (19.8)	1.13 (0.88, 1.44)	0.35
SAEs (%)	263 (46.1)	277 (46)	0.92 (0.71, 1.18)	0.50
EQ-5D HUS (/1)	0.34 (0.39)	0.37 (0.4)	−0.01 (−0.05, 0.03)	0.65
EQ-VAS (/100)	48.1 (34.5)	49.9 (33.1)	0.53 (−2.63, 3.69)	0.74
Discharge				
Length of hospital stay, days	62.3 (47.6)	63.2 (48.5)	1.72 (−3.81, 7.24)	0.54
Death by discharge (%)	103 (18)	98 (16.3)	1.02 (0.72, 1.44)	0.93

Treatment effect of tranexamic acid versus placebo assessed using Cox proportional hazards, multiple linear, binary or ordinal logistic regression with adjustment for baseline prognostic factors.

Bold p-values indicate <0.05

*Also adjusted for baseline haematoma volume.

cOR, common OR; EQ-VAS, EuroQoL Visual Analogue Scale; EQ-5D HUS, EuroQoL-5 Dimensions Health Utility Status; HV, haematoma volume; MD, mean difference; mRS, modified Rankin Scale; NIHSS, National Institutes of Health Stroke Scale; SAEs, serious adverse events; SBP, systolic blood pressure.

**Figure 1:**
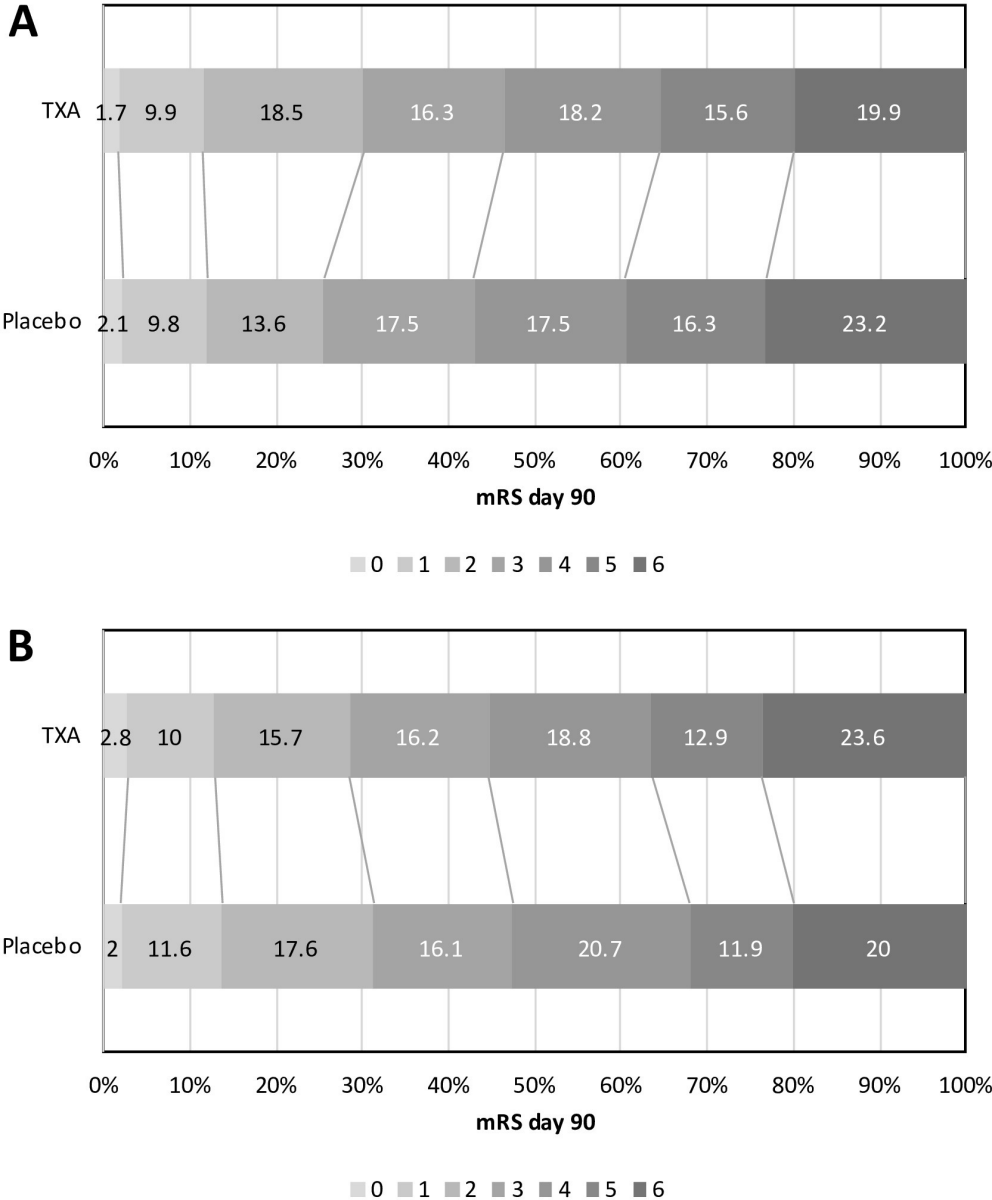
Effect of TXA versus placebo on the primary outcome (mRS) by prerandomisation baseline systolic blood pressure. (A) Distribution of mRS at day 90 by TXA versus placebo in those with baseline systolic BP≤170 mm Hg: cOR 0.73, 95% CI 0.59 to 0.91, p=0.005. Ordinal logistic regression with adjustment for baseline prognostic factors. (B) Distribution of mRS at day 90 by TXA versus placebo in those with baseline systolic BP>170 mm Hg: cOR 1.05, 95% CI 0.85 to 1.30, p=0.63. Ordinal logistic regression with adjustment for baseline prognostic factors. BP, blood pressure; cOR, common OR; mRS, modified Rankin Scale; TXA, tranexamic acid.

At day 90, in those with baseline ≤SBP 170 mm Hg, the primary outcome demonstrated a favourable shift to less death and dependency in those randomised to tranexamic acid compared with placebo (cOR 0.73, 95% CI 0.59 to 0.91, figure 1A). This finding was not altered after also adjusting for baseline haematoma volume and haematoma location. In those with baseline SBP≤170 mm Hg, there was a significant interaction between haematoma location and treatment with tranexamic acid on the primary outcome (p=0.012). The interaction term for those with baseline SBP>170 mm Hg was non-significant. Improved quality of life scores were seen in participants randomised to tranexamic acid (table 2).

In contrast, there were no significant treatment effects of tranexamic acid in those participants with baseline SBP>170 mm Hg (figure 1B, table 2).

### Outcomes by change in SBP

Those with >15% decrease in SBP from baseline to day 2 had a higher baseline SBP than the 5%–15% decrease, reference (5% decrease to 5% increase) and >5% increase groups. There was a trend to lower baseline SBP across the groups: p<0.001 (table 3).

**Table 3:** Associations between change in systolic BP from baseline to day 2 and clinical outcomes

Overall	>15% decrease		5%–15% decrease		5% decrease to 5% increase	>5% increase	
	cOR/OR/HR/MD (95% CI)	P value	cOR/OR/HR/MD (95% CI)	P value	(Reference)	cOR/OR/HR/MD (95% CI)	P value
N (%)	994		601		401	230	
Baseline systolic BP, mm Hg, mean (SD)	188.1 (24.6)		167.8 (20.5)		157.8 (20.2)	143.5 (19.9)	
Haematoma							
Change in volume from baseline to 24 hours*, mL	−0.58 (-3.07, 1.92)	1.00	−0.27 (-2.73, 2.19)	1.00	0	−0.73 (-2.46, 3.92)	1.00
Haematoma expansion (%)	0.95 (0.69, 1.29)	0.72	0.83 (0.61, 1.13)	0.23	1	1.04 (0.71, 1.54)	0.83
Day 2							
Death (%)	0.58 (0.24, 1.42)	0.24	0.48 (0.18, 1.25)	0.13	1	1.54 (0.60, 3.94)	0.37
SAEs (%)	1.22 (0.91, 1.64)	0.18	0.97 (0.72, 1.30)	0.83	1	1.34 (0.93, 1.93)	0.12
Day 7							
Death (%)	0.71 (0.42, 1.19)	0.19	0.43 (0.24, 0.76)	**0.003**	1	1.78 (1.02, 3.12)	**0.044**
SAEs (%)	1.26 (0.95, 1.68)	0.11	1.05 (0.79, 1.40)	0.72	1	1.55 (1.08, 2.22)	**0.018**
Day 90							
Primary outcome							
mRS [/6]	1.07 (0.84, 1.35)	0.59	1.00 (0.79, 1.27)	0.97	1	1.34 (0.99, 1.81)	0.063
Death (%)	0.79 (0.59, 1.06)	0.11	0.83 (0.62, 1.11)	0.21	1	1.08 (0.77, 1.53)	0.66
SAEs (%)	1.21 (0.91, 1.62)	0.19	1.03 (0.78, 1.37)	0.82	1	1.35 (0.94, 1.95)	0.11
Tranexamic acid, N (%)	491		293		210	122	
Baseline systolic BP, mm Hg, mean (SD)	187.6 (24.8)		166.6 (20.5)		158 (21.7)	142.2 (19.2)	
Haematoma							
Change in volume from baseline to 24 hours*, mL	−1.81 (-5.21, 1.58)	0.95	−2.15 (-5.51, 1.21)	0.55	0	−0.22 (-4.48, 4.03)	1.00
Haematoma expansion (%)	0.99 (0.63, 1.55)	0.95	0.91 (0.58, 1.43)	0.68	1	1.01 (0.58, 1.77)	0.97
Day 2							
Death (%)	0.32 (0.09, 1.08)	0.07	0.35 (0.09, 1.46)	0.15	1	2.13 (0.62, 7.31)	0.23
SAEs (%)	1.24 (0.82, 1.87)	0.32	0.97 (0.64, 1.47)	0.88	1	1.13 (0.67, 1.89)	0.65
Day 7							
Death (%)	0.41 (0.20, 0.85)	**0.017**	0.37 (0.16, 0.82)	**0.015**	1	1.87 (0.85, 4.12)	0.12
SAEs (%)	1.19 (0.80, 1.77)	0.40	0.83 (0.56, 1.24)	0.37	1	1.18 (0.72, 1.94)	0.52
Day 90							
Primary outcome							
mRS [/6]	0.95 (0.68, 1.32)	0.76	1.08 (0.78, 1.50)	0.64	1	1.35 (0.89, 2.06)	0.16
Death (%)	0.62 (0.42, 0.93)	**0.022**	0.75 (0.49, 1.13)	0.17	1	0.99 (0.61, 1.60)	0.97
SAEs (%)	1.09 (0.73, 1.63)	0.67	0.84 (0.56, 1.24)	0.37	1	0.89 (0.54, 1.46)	0.64
Placebo, N (%)	503		308		191	108	
Baseline systolic BP, mm Hg, mean (SD)	188.6 (24.3)		168.9 (20.5)		157.7 (18.4)	144.9 (20.7)	
Haematoma							
Change in volume from baseline to 24 hours*, mL	0.60 (-3.07, 4.28)	1.00	1.42 (-2.19, 5.03)	1.00	0	1.49 (-3.33, 6.32)	1.00
Haematoma expansion (%)	0.92 (0.59, 1.42)	0.69	0.76 (0.49, 1.16)	0.20	1	1.05 (0.60, 1.83)	0.87
Day 2							
Death (%)	1.35 (0.34, 5.30)	0.67	0.66 (0.16, 2.77)	0.57	1	1.04 (0.21, 5.22)	0.96
SAEs (%)	1.21 (0.79, 1.84)	0.38	0.97 (0.64, 1.46)	0.87	1	1.54 (0.91, 2.61)	0.11
Day 7							
Death (%)	1.21 (0.57, 2.58)	0.62	0.52 (0.23, 1.18)	0.12	1	1.78 (0.78, 4.06)	0.17
SAEs (%)	1.36 (0.90, 2.06)	0.15	1.33 (0.88, 2.00)	0.17	1	1.99 (1.17, 3.39)	**0.011**
Day 90							
Primary outcome							
mRS [/6]	1.18 (0.84, 1.65)	0.35	0.91 (0.65, 1.27)	0.56	1	1.32 (0.85, 2.07)	0.22
Death (%)	0.98 (0.64, 1.52)	0.94	0.81 (0.52, 1.26)	0.34	1	0.98 (0.58, 1.64)	0.93
SAEs (%)	1.37 (0.90, 2.08)	0.14	1.28 (0.85, 1.93)	0.24	1	2.14 (1.24, 3.70)	**0.007**

Ordinal, binary, multiple linear or Cox regression with adjustment for baseline prognostic factors. The 5% decrease to 5% increase group is the reference group for comparisons.

Bold p-values indicate <0.05

*Adjusted for baseline haematoma volume.

BP, blood pressure; cOR, common OR; MD, mean difference; mRS, modified Rankin Scale; NIHSS, National Institutes of Health Stroke Scale; SAEs, serious adverse events.

Overall, compared with the reference group (5% decrease to 5% increase), a >5% increase in SBP from baseline to day 2 was associated with increased death and SAEs at day 7. When assessed within treatment groups, a >5% increase in SBP from baseline to day 2 in those randomised to placebo was associated with increased SAEs at days 7 and 90; an effect not seen in those randomised to tranexamic acid (table 3).

Overall, a >15% decrease in SBP from baseline to day 2 was associated with fewer deaths by day 7 and day 90; the same effect was seen in those randomised to tranexamic acid, but not in participants randomised to placebo (table 3).

There were fewer deaths at day 7 in those participants with a 5%–15% decrease in SBP from baseline to day 2 compared with the reference group, both overall and in those randomised to tranexamic acid (table 3).

By day 2, 1541 (66.3%) participants’ SBP was over 140 mm Hg. In an on-treatment analysis, there was no difference in clinical outcomes between treatment groups when assessed by day 2 SBP≤140 mm Hg vs >140 mm Hg or across the spectrum of day 2 SBP (online supplemental figure 1)

### BP lowering treatments

Of 2325 participants in the trial, 1736 (74.9%) received any BP lowering therapy by day 2 with a greater proportion receiving any treatment with baseline SBP>170 mm Hg compared with those with baseline SBP≤170 mm Hg: 89.2% vs 60.5%, p<0.001 (online supplemental table 3). The median number of agents used was 2 [1, 3] and 1 [0, 2] in those with baseline SBP>170 mm Hg and≤170 mm Hg, respectively. Intravenous then oral routes were the most commonly used with 74.8% of those with baseline SBP>170 mm Hg receiving an intravenous agent. Overall, the most popular classes of agents were as follows: β-blocker (including labetalol) 39.9%; calcium channel blocker 32.2%; nitrate 59.2%. The number, route and class of antihypertensive agents did not differ between tranexamic acid and placebo groups (data not shown). Overall, those participants treated with either a calcium channel blocker or ACE inhibitor (ACEi) had a shift to less death and dependency at day 90 compared with those who did not receive these medications: calcium channel blockers cOR 0.82, 95% CI 0.69 to 0.98; ACEi cOR 0.75, 95% CI 0.61 to 0.92. No associations were seen for other antihypertensive drug groups.

Over the course of the trial, the use of BP lowering therapy by day 2 increased from 33.3% in quarter 2 of 2013 to 81.8% in quarter 4 of 2017 (χ^2^ and Mantel-Haenszel test for trend p<0.001, online supplemental figure 2).

## Discussion

In this prespecified secondary analysis of the TICH-2 trial, randomisation to tranexamic acid in the presence of baseline SBP≤170 mmHg was associated with less haematoma expansion and improved clinical outcomes with fewer deaths and SAEs, less death and dependency, and improved quality of life scores compared with placebo. This was despite patients being older, randomised later and having larger baseline haematoma volumes than those with SBP>170 mm Hg. A>15% reduction in SBP from baseline to day 2 was associated with fewer deaths by day 7 and 90 in those randomised to tranexamic acid but not placebo, while a>5% increase in SBP was associated with increased death and SAEs by day 7 overall, and increased SAEs in those randomised to placebo by day 7 and 90. BP lowering treatment by day 2 was more frequently used over the time course of the trial in line with changes in international clinical guidelines. Despite this, 66% of the trial population remained hypertensive on day 2.

Baseline characteristics differed by baseline SBP group; participants with SBP≤170 mm Hg had larger haematomas in lobar locations on average, while participants with SBP>170 mm Hg had deep haematomas on a background of hypertension. This may, in part, reflect the distribution of ICH due to underlying aetiology, that is, lobar haematomas in CAA and deep haematomas secondary to hypertensive arteriolopathy. A multicentre cohort study in China involving 5656 patients with ICH found that admission BP differed by ICH aetiology with patients with CAA having a lower BP and larger haematoma volumes than patients with presumed hypertensive arteriolopathy who had a higher BP and smaller haematoma volumes at baseline.^17^ Dichotomising ICH location as either lobar or non-lobar may risk over-simplifying the underlying aetiology given that lobar ICH comprises CAA-related ICH, hypertensive arteriolopathy and mixed cerebral small vessel disease.^18^ Recently, a detailed secondary imaging analysis of TICH-2 demonstrated that in participants with lobar CAA-related ICH, there was an increased risk of haematoma expansion with increasing time from randomisation, while the risk of haematoma expansion was constant irrespective of baseline haematoma volume.^19^ These effects were not seen in those with non-CAA lobar or non-lobar ICH and may suggest a difference in haematoma dynamics between these ICH groups.^19^ CAA-related bleeding may originate from leptomeningeal vessels and have more space to expand into (including the subarachnoid space), resulting in prolonged, slower, lower pressure bleeding over several hours.^19^ This may potentially provide a longer treatment window for haemostatic agents including tranexamic acid to exert their effects. These hypotheses require further testing and should be considered in ongoing and future studies of tranexamic acid in ICH.

Although trials to date have yet to demonstrate a positive effect of haemostatic therapies on clinical outcome in ICH,^20^ there are several possible explanations for tranexamic acid being associated with improved clinical outcomes in those with baseline SBP≤170 mm Hg. First, a lower baseline SBP is associated with less haematoma expansion than higher baseline SBP,^1–3^ therefore, any potential treatment effect of tranexamic acid on haematoma expansion may be larger as there is no separate pathological mechanism in the form of elevated BP to overcome. However, in the present analysis, there was no difference in the rate of haematoma expansion between baseline SBP groups: 28.2% in the≤170 mm Hg group vs 25.7% in the >170 mm Hg group, p=0.21. Second, by dichotomising baseline SBP, we may have unintentionally selected a group of participants more likely to benefit from tranexamic acid independent of BP, such as people with moderate-sized lobar haematomas as opposed to smaller deep haematomas. Although sensitivity analyses adjusting for baseline haematoma characteristics did not alter the treatment effect of tranexamic acid, there was a significant interaction between haematoma location and tranexamic acid on the primary outcome in those with baseline SBP≤170 mm Hg suggesting that there may be a differential effect of tranexamic acid depending on haematoma location.

Those participants with a >15% decrease in SBP from baseline to day 2 were less likely to die by days 7 and 90 when randomised to tranexamic acid; a finding not seen in those randomised to placebo. In contrast, a >5% increase in SBP from baseline to day 2 was associated with increased death and SAEs at day 7 overall, and increased SAEs at days 7 and 90 in those randomised to placebo. Therefore, a reduction in BP from baseline to day 2 combined with treatment with tranexamic acid may be beneficial, while an increase in BP in those randomised to placebo may be harmful. These results should be considered preliminary given their observational nature within a randomised controlled trial. However, one plausible explanation for why BP lowering and tranexamic acid may be additive or even synergistic in ICH is by attenuating haematoma expansion and improving clinical outcome.

The strengths of this study include the prespecified nature of the analyses within the context of the largest trial of haemostatic therapies in ICH with almost complete follow-up data. However, there are several limitations. First, we do not have data on what BP lowering medications patients were taking prior to their ICH. Therefore, we were unable to establish any association between the use of pre-ICH antihypertensives and prerandomisation baseline BP. This includes any BP lowering medication given after symptom onset of their ICH but before randomisation, which may have influenced the baseline BP recorded prior to randomisation. Second, there were few BP readings recorded in the trial; two measurements prerandomisation and two measurements on day 2. Therefore, we were unable to assess any effect of BP variability, which has been shown to be a stronger predictor of clinical outcome in acute ICH^21 22^ and ischaemic stroke^23^ than absolute BP. Nor could we adjust for early BP change within the first hours after ICH. Instead, we used the change in BP from baseline to day 2 to look for any associations with outcome. These analyses were within randomised treatment groups, are therefore observational, may represent chance and should be considered as hypothesis generating. Third, ICH aetiology was investigator-reported and largely determined by CT imaging. This may have led to less precise characterisation of ICH aetiology, particularly in those with mixed pathology. Last, given this is a subgroup analysis, our findings may represent chance. Although we did not adjust for multiplicity of testing, this subgroup analysis was prespecified in the statistical analysis plan of the main TICH-2 trial (including the SBP cut-point), has biological plausibility, had a positive interaction with treatment on outcome and followed the statistical analysis plan of the main TICH-2 trial.

In summary, this prespecified subgroup analysis of the TICH-2 trial demonstrated that randomisation to tranexamic acid in participants with baseline SBP≤170 mm Hg was associated with less haematoma expansion and improved clinical outcomes across multiple domains both early and late after ICH. Future research should seek to establish which subgroups of patients may benefit from tranexamic acid in acute ICH, including by haemorrhage location, size and underlying aetiology including CAA. Whether BP lowering is additive or synergistic in the presence of tranexamic acid in acute ICH is unclear and future clinical trials may help to provide a clearer treatment paradigm for clinicians.

## Data Availability

Data are available on reasonable request. Data pertaining to this manuscript are available from the corresponding author on reasonable request.
