# Supplementary material for: Effects of blood pressure and tranexamic acid in spontaneous intracerebral haemorrhage: a secondary analysis of a large randomised controlled trial
Source: BMJ Neurol Open. 2023 Jun 12;5(1):e000423. doi: 10.1136/bmjno-2023-000423 (PMC10277112; doi:10.1136/bmjno-2023-000423)
Supplement: Supplementary data [file bmjno-2023-000423supp001.pdf]

SUPPLMENTARY MATERIAL

TABLE OF CONTENTS

	Page
Title	1
List of supplementary Tables and Figures	*2*
Tables (start from)	*3*
Figures (start from)	*6*

Title
The effects of blood pressure and tranexamic acid in spontaneous intracerebral haemorrhage: a secondary analysis of a large randomised controlled trial

Appleton JP, *et al. BMJ Neurol Open* 2023; 5:e000423. doi: 10.1136/bmjno-2023-000423

LIST OF SUPPLEMENTARY TABLES AND FIGURES

	Title	Page
**Tables**		
1	*Investigator-reported final diagnosis by pre-randomisation baseline systolic blood pressure*	*3*
2	*Sub-categories of serious adverse events by randomised treatment and pre-randomisation baseline systolic blood pressure*	*4*
3	*Blood pressure lowering treatments post-randomisation split by pre-randomisation systolic blood pressure*	*5*
**Figures**		
1	*Effect of tranexamic acid vs. placebo on primary outcome (mRS) by day 2 systolic blood pressure in 20 mmHg increments*	*6*
2	*Blood pressure lowering therapy between randomisation and day 2, over the course of the trial*	*7*

TABLES

Supplementary Table 1: Investigator-reported final diagnosis by pre-randomisation baseline systolic blood pressure.
Data are number (%); comparison across groups by Chi-square test

	All	SBP ≤170 mmHg	SBP >170 mmHg	p
Intracerebral haemorrhage with no underlying cause (%)	1019 (43.8)	583 (50.6)	436 (37.2)	**<0.001**
Intracerebral haemorrhage with underlying cause (%)	1289 (55.4)	559 (48.5)	730 (62.2)	**<0.001**
Cerebral amyloid angiopathy	80 (3.4)	58 (5.0)	22 (1.9)	**<0.001**
Arteriovenous malformation (AVM)	25 (1.1)	15 (1.3)	10 (0.9)	0.32
Tumour	5 (0.2)	4 (0.3)	1 (0.1)	0.21
Aneurysm	13 (0.6)	8 (0.7)	5 (0.4)	0.42
Venous infarct	5 (0.2)	3 (0.3)	2 (0.2)	0.68
Coagulopathy	25 (1.1)	15 (1.3)	10 (0.9)	0.07
Hypertensive arteriopathy	1121 (48.2)	449 (39.0)	672 (57.3)	**<0.001**
Other	33 (1.4)	16 (1.4)	17 (1.4)	0.99
Ischaemic stroke with haemorrhagic transformation (%)	5 (0.2)	3 (0.3)	2 (0.2)	0.68
Non-stroke/other (%)	11 (0.5)	7 (0.6)	4 (0.3)	0.38

SBP: systolic blood pressure

Appleton JP, *et al. BMJ Neurol Open* 2023; 5:e000423. doi: 10.1136/bmjno-2023-000423

Supplementary Table 2: Sub-categories of serious adverse events by randomised treatment and pre-randomisation baseline systolic blood pressure

Data are number (%); comparison across randomised treatment groups by Chi-square test

	All	Tranexamic acid	Placebo	p
**SBP ≤170 mmHg**	1152	590	562	
Nervous system disorders	391 (33.9)	180 (30.5)	211 (37.5)	**0.012**
Cardiac disorders	70 (6.1)	38 (6.4)	32 (5.7)	0.60
Respiratory disorders	156 (13.5)	67 (11.4)	89 (15.8)	**0.026**
Gastrointestinal disorders	15 (1.3)	7 (1.2)	8 (1.4)	0.72
Genitourinary disorders	12 (1)	6 (1)	6 (1.1)	0.93
Haematological disorders	4 (0.3)	2 (0.3)	2 (0.4)	0.96
Musculoskeletal disorders	12 (1)	6 (1)	6 (1.1)	0.93
Miscellaneous	25 (2.2)	18 (3.1)	7 (1.2)	**0.036**
**SBP >170 mmHg**	1173	571	602	
Nervous system disorders	394 (33.6)	195 (34.2)	199 (33.1)	0.69
Cardiac disorders	60 (5.1)	31 (5.4)	29 (4.8)	0.64
Respiratory disorders	141 (12)	65 (11.4)	76 (12.6)	0.51
Gastrointestinal disorders	9 (0.8)	5 (0.9)	4 (0.7)	0.68
Genitourinary disorders	23 (2)	11 (1.9)	12 (2)	0.93
Haematological disorders	2 (0.2)	0	2 (0.3)	0.17
Musculoskeletal disorders	12 (1)	5 (0.9)	7 (1.2)	0.63
Miscellaneous	27 (2.3)	14 (2.5)	13 (2.2)	0.74

Appleton JP, *et al. BMJ Neurol Open* 2023; 5:e000423. doi: 10.1136/bmjno-2023-000423

Supplementary Table 3: Blood pressure lowering treatments post-randomisation split by pre-randomisation baseline systolic blood pressure. Data are number (%); comparison across randomised treatment groups by Chi-square test or Kruskal-Wallis test

	**Overall**	**SBP ≤170 mmHg**	**SBP >170 mmHg**	**p**
BP lowering drugs used, yes (%)	1736 (74.9)	696 (60.5)	1040 (89.2)	<0.001
No. of BP lowering drugs used, median [IQR]	1 [0,2]	1 [0,2]	2 [1,3]	<0.001
No. of BP lowering drugs used (%)				<0.001
0	581 (25.1)	455 (39.5)	126 (10.8)	
1	678 (29.3)	317 (27.5)	361 (31)	
2	561 (24.2)	207 (18)	354 (30.4)	
3	320 (13.8)	114 (9.9)	206 (17.7)	
4	121 (5.2)	45 (3.9)	76 (6.5)	
5	42 (1.8)	8 (0.7)	34 (2.9)	
Route of BP lowering drug (%)				
Oral	952 (40.9)	410 (35.6)	542 (46.2)	<0.001
Nasogastric	90 (3.9)	36 (3.1)	54 (4.6)	0.07
Intravenous	1299 (55.9)	422 (36.6)	877 (74.8)	<0.001
Patch	235 (10.1)	93 (8.1)	142 (12.1)	0.001
Class of antihypertensive used (%)				
ACEi	444 (19.1)	190 (16.5)	254 (21.7)	0.002
ARB	100 (4.3)	45 (3.9)	55 (4.7)	0.35
α-blocker	157 (6.8)	62 (5.4)	95 (8.1)	0.009
β-blocker (including labetalol)	927 (39.9)	316 (27.4)	611 (52.1)	<0.001
Calcium channel blocker	749 (32.2)	291 (25.3)	458 (39)	<0.001
Diuretic	295 (12.7)	120 (10.4)	175 (14.9)	0.001
Glyceryl trinitrate	678 (29.2)	222 (19.3)	456 (38.9)	<0.001
Nitrate	697 (30)	230 (20)	467 (39.8)	<0.001

ACEi: angiotensin converting enzyme inhibitor; ARB: angiotensin receptor blocker; BP: blood pressure; IQR: interquartile range; SBP: systolic blood pressure.

FIGURES

Supplementary Figure 1: Effect of tranexamic acid vs. placebo on primary outcome (mRS) by day 2 systolic blood pressure in 20 mmHg increments

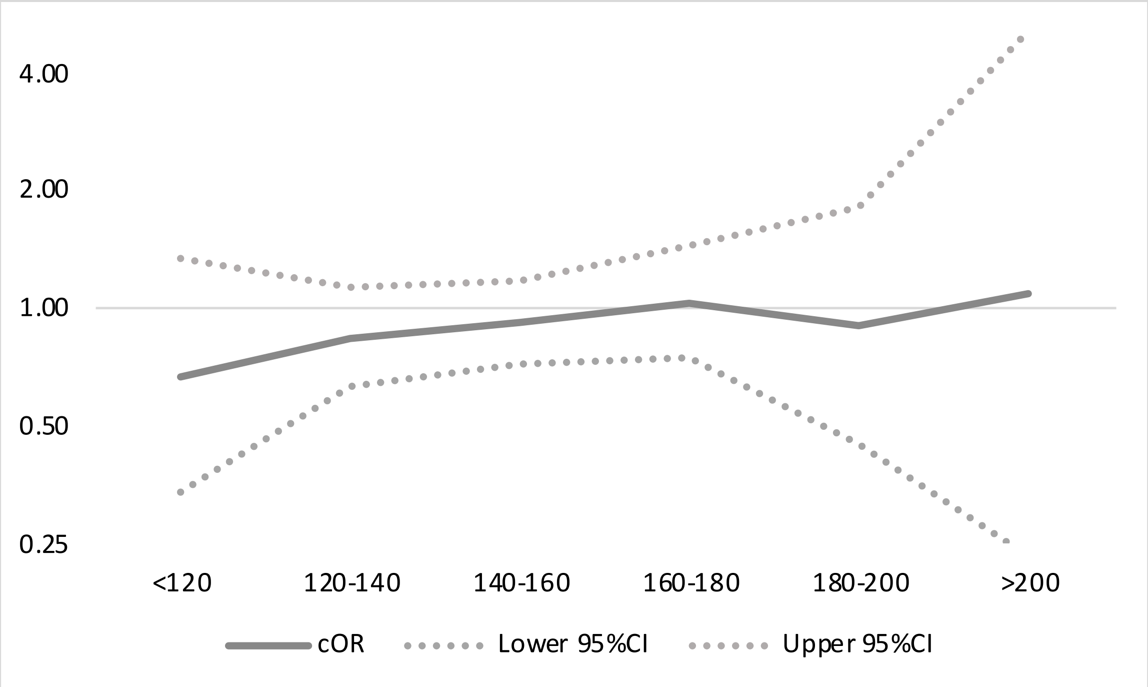

Ordinal logistic regression with adjustment for prognostic variables. CI: confidence interval; cOR: common odds ratio; mRS: modified Rankin Scale.

Appleton JP, *et al. BMJ Neurol Open* 2023; 5:e000423. doi: 10.1136/bmjno-2023-000423

Supplementary Figure 2: Blood pressure lowering therapy between randomisation and day 2, over the course of the trial

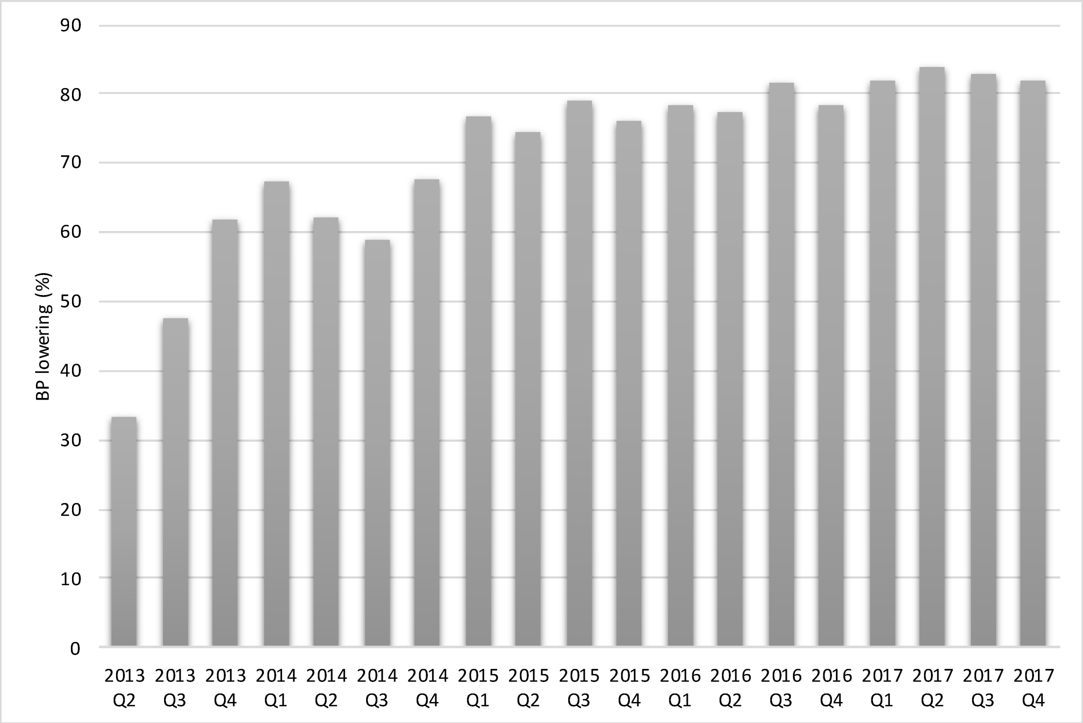

% of patients receiving BP lowering therapy on day 2 by quarter of recruitment throughout the trial. X^2 $p<0.001$ and Mantel-Haenszel test for trend $p<0.001$ for change in BP lowering over time

Appleton JP, *et al. BMJ Neurol Open* 2023; 5:e000423. doi: 10.1136/bmjno-2023-000423
